# Abnormal coordination of upper extremity during target reaching in persons post stroke

**DOI:** 10.1038/s41598-023-39684-4

**Published:** 2023-08-08

**Authors:** Kyung Koh, Giovanni Oppizzi, Glenn Kehs, Li-Qun Zhang

**Affiliations:** 1 Fischell Department of Bioengineering, University of Maryland, College Park, MD 20742 USA; 2 Department of Physical Therapy and Rehabilitation Science, University of Maryland, 100 Penn St, Baltimore, MD 21201 USA; 3 University of Maryland Rehabilitation and Orthopaedic Institute, Baltimore, MD 21207 USA; 4 Department of Neurology, University of Maryland, Baltimore, MD 21201 USA; 5 Department of Orthopaedics, University of Maryland, Baltimore, MD 21201 USA

**Keywords:** Stroke, Motor control, Engineering

## Abstract

Understanding abnormal synergy of the upper extremity (UE) in stroke survivors is critical for better identification of motor impairment. Here, we investigated to what extent stroke survivors retain the ability to coordinate multiple joints of the arm during a reaching task. Using an exoskeleton robot, 37 stroke survivors’ arm joint angles (θ) and torques (τ) during hand reaching in the horizontal plane was compared to that of 13 healthy controls. Kinematic and kinetic coordination patterns were quantified as variances of the multiple-joint angles and multiple-joint torques across trials, respectively, that were partitioned into task-irrelevant variance (*TIV*_*θ*_ and *TIV*_*τ*_) and task-relevant variance (*TRV*_*θ*_ and *TRV*_*τ*_). *TIV*_*θ*_ and *TRV*_*θ*_ (or *TIV*_*τ*_ and *TRV*_*τ*_) led to consistent and inconsistent hand position (or force), respectively. The index of synergy (*IS*_θ_ and *IS*_τ_) was determined as ${IS}_{\theta }=({TIV}_{\theta }-{TRV}_{\theta })/({TIV}_{\theta }+{TRV}_{\theta })$ and ${IS}_{\tau }=({TIV}_{\tau }-{TRV}_{\tau })/({TIV}_{\tau }+{TRV}_{\tau })$ for kinematic and kinetic coordination patterns, respectively. Both kinematic *IS*_θ_ and kinetic *IS*_τ_ in the stroke group were significantly lower than that of the control group, indicating stroke survivors had impaired reaching abilities in utilizing the multiple joints of the UE for successful completion of a reaching task. The reduction of kinematic *IS*_θ_ in the stroke group was mainly attributed to the lower *TIV*_*θ*_ as compared to the control group, while the reduction of kinetic *IS*_τ_ was mainly due to the higher ${TRV}_{\tau }$ as well as lower *TIV*_τ_. Our results also indicated that stroke may lead to motor deficits in formation of abnormal kinetic synergistic movement of UE, especially during outward movement. The findings in abnormal synergy patterns provides a better understanding of motor impairment, suggesting that impairment-specific treatment could be identified to help improve UE synergies, focusing on outward movements.

## Introduction

Humans are capable of robust control of the upper extremity (UE) in which dynamic precision control of multiple body segments is required. To achieve this remarkable control, the central nervous system (CNS) is required to coordinate multiple body segments for the stabilization of the overall action of certain tasks. Motor synergy has been proposed as a control mechanism of the CNS^1^, referred to as specific patterns of the multi-degrees of freedoms (DoF) movement in completing motor tasks^2–4^. Cumulating evidence on postural control indicates that the CNS is able to synergistically control multiple DoFs such as multiple segments, multiple muscles, and multiple fingers for the stabilization of their combined actions such as controlling the body center of mass^5,6^, multiple muscle activations^7,8^, and multi-finger grasping^9–11^.

After neurological disorders such as stroke, the synergistic patterns of upper extremity (UE) movements are known to be altered^12–14^. While the term "synergy" has traditionally been used clinically to describe coordination deficits characterized by abnormal muscle co-activation following stroke, recent research in the field of neuroscience has indicated that motor synergy refers to task-specific coordinated patterns of movement involving multiple degrees of freedom (DoFs)^2,3,15^. These synergistic patterns are believed to be fundamental aspects of the CNS's control mechanisms for coordinating multiple DoFs in a way that stabilizes or improves performance during specific tasks.

Although several studies have investigated changes in kinematic motor synergies of upper extremity movements in individuals with stroke, the findings have been inconsistent^16–18^. Gera, et al.^16^ reported diminished interjoint coordination in stroke survivors, which could affect hand path consistency. On the other hand, Reisman and Scholz^17^ found that some aspects of joint coordination were preserved during pointing tasks in individuals with post-stroke hemiparesis. However, the inconsistent findings across these studies underscore the need for further systematic investigations to examine the effects of stroke on both the kinematics and kinetics of motor synergies during upper extremity movements. Understanding these changes is crucial for accurate identification of motor impairments and the development of effective rehabilitation strategies.

Recently, advanced rehabilitation robotics have emerged as a valuable tool for high-intensity, repetitive, and task-specific treatment of impaired upper limbs, as well as for the systematic assessment of multi-joint movements and synergy^19–22^. In line with this, our previous work ^23–2^ focused on the development of a multi-joint rehabilitation robot named IntelliArm. This innovative system was specifically designed to assess the dynamics of the shoulder, elbow, and wrist individually and simultaneously. By utilizing the IntelliArm, we were able to achieve quantitative and systematic characterizations of neuromuscular changes across multiple joints. The integration of robotics in rehabilitation not only provides a controlled and standardized environment but also enables objective measurements of kinetic and kinematic variables, enhancing our understanding of motor synergies and facilitating tailored interventions for stroke survivors.

Here, we investigated how stroke affects synergistic patterns during a target reaching task. Two targets were presented: one that can be reached with inward motion including shoulder horizontal adduction, elbow flexion, and wrist flexion, and a second target that can be reached with outward motion including shoulder horizontal abduction, elbow extension, and wrist extension. We hypothesized that (1) individuals post stroke would demonstrate impaired synergistic patterns both in kinematics and kinetics as compared to healthy controls, and that (2) the expected impairment of synergy in stroke survivors would be more severe during outward movement (i.e., away from the body) as compared to inward movement (i.e., toward the body) considering stroke is often accompanied by flexor hypertonia of UE.

## Methods

### Participants

Thirty-seven stroke survivors and thirteen age-matched control subjects were recruited for this study. All the control subjects were right-handed (6 males and 7 females, mean age 48.5 years, SD 15.8). Twenty-one stroke survivors were right hemiparetic, and the other 16 survivors were left hemiparetic (27 males and 10 females, mean age 59.1 years, SD 12.7). The difference in sample sizes between the control and stroke groups was due to the specific characteristics of our study population. The larger sample size in the post-stroke group helped account for the increased variability commonly observed in stroke survivors, which can be influenced by factors such as lesion location, severity, and individual recovery trajectories. This increased variability in the post-stroke group warranted a larger sample size to ensure the robustness and generalizability of our findings. It is important to note that the primary focus of our study was to investigate the effects of stroke on upper limb motor function, and the control group served as a reference for comparison. The inclusion criteria of the stroke patients were (1) first focal unilateral lesion, ischemic or hemorrhagic; (2) at least six months post stroke; (3) had cognitive ability to follow simple instructions; (4) ability to provide informed consent. Individuals were excluded if they had: (1) apraxia; (2) severe cardiovascular conditions; (3) unrelated musculoskeletal injuries. Prior to the experiment, each participant gave a written informed consent, and the study is approved by the institutional review board of the University of Maryland, Baltimore. All methods were carried out in accordance with relevant guidelines and regulations.

### Assessment of upper limb motor function

Assessment of upper limb motor function was conducted using the Fugl-Meyer Assessment of upper limb (FMA-UE)^23^. The FMA-UE is a widely used and reliable measure of upper limb motor function, with a higher score indicating better motor function. It comprises various subsections, including a separate section for hand function. In this study, FMA-UE was administered by trained assessors who were blinded to the participants' group assignments.

### Experiments

Prior to the measurement trial, all subjects performed a practice session consisting of reaching movements to each target. This practice session aimed to familiarize the subjects with the task requirements and minimize any learning effects during the actual measurement. During the practice session, subjects were instructed to perform the reaching movements at a self-selected comfortable pace. They were encouraged to focus on accuracy and consistency of the movement while maintaining a natural and fluid motion. Instructions regarding the movement duration were provided to ensure that subjects understood the task requirements but without imposing strict timing constraints. Specifically, subjects were instructed to perform the reaching movements without rushing or delaying the movement, aiming for a smooth and continuous motion from the starting position to the target.

**Figure 1:**
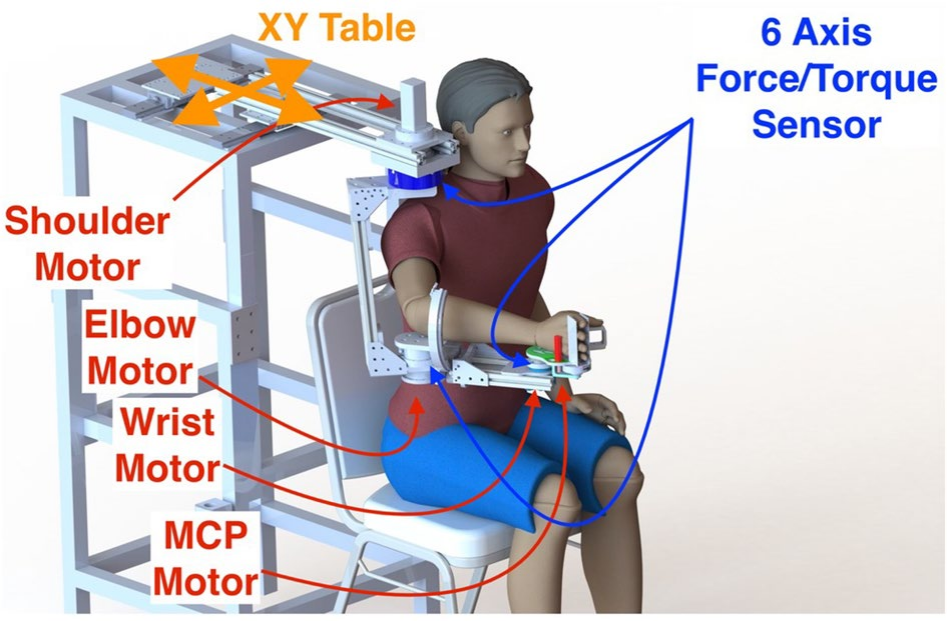
A multi-joint rehabilitation robot, called IntelliArm, controls the shoulder, elbow, and wrist movement simultaneously. The shoulder horizontal abduction/adduction, and elbow and wrist flexion/extension are controlled by the shoulder, elbow, and wrist motor, respectively. The robotic arm is mounted on a large X–Y table, keeping the robot shoulder axis aligned with the shoulder horizontal adduction axis of the subject.

In order to assess UE function, we used a robot for neurorehabilitation of the shoulder elbow, and wrist joints we had previously developed. The robot called IntelliArm was designed to perform a two-dimensional motion on the horizontal plane with gravity support of UE. All subjects were asked to sit upright comfortably, and to mount the upper arm, forearm and hand to the IntelliArm (Fig. 1). The robot arm lengths for the upper arm and forearm were then adjusted to align mechanical axes for shoulder horizontal adduction/abduction, and elbow and wrist flexion/extension with the corresponding subject’s anatomical joint axes. The subject’s upper arm and forearm were strapped to the corresponding braces to ensure proper alignment throughout the experiment. Initial position was at shoulder horizontal adduction of 70°, elbow flexion of 60° and wrist flexion of 0°, respectively.

**Figure 2:**
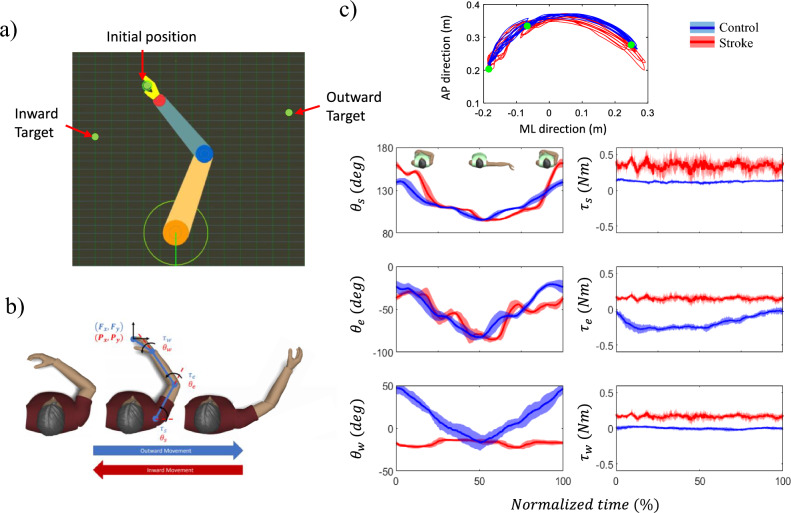
Virtual display of subject’s upper extremity with the multiple targets (**a**). Joint angles and torques of representative subjects of the control (blue) and the stroke (red) (**b**). The mean (solid line) and one standard deviation (shaded area) of trajectories of the end-effector positions, joint angles and torques across trials were presented during a target-reaching task (**c**). The green circles represent the inward and outward targets displayed during the task.

The IntelliArm was made backdrivable by realizing a low impedance through robust impedance control so that subjects can move the arm with the IntelliArm while only feeling a low inertia^24^. The subject’s virtual UE and two target points were displayed on a 50″ TV located about one meter from the subject. The subject was asked to move the hand from the initial position to Target 1 and to Target 2 (Fig. 2a). The subject was asked to follow this sequence for 10 repetitions. A circle in the virtual hand needs to overlap the green-dot target for a successful match (Fig. 2a). Final position error was calculated as the distance between the center of circle and the target position when the circle is closest to target.

### Uncontrolled manifold analysis

We employed the uncontrolled manifold analysis (UCM) to quantify how the subject utilizes multiple degrees of freedom of their upper extremity (UE) during a reaching target task. It has been extensively used to investigate kinematic synergies, such as joint angles, during various motor tasks^25,26^. The UCM framework allows for the quantification of joint angle variability across multiple trials relative to the desired task outcome, such as fingertip trajectories during reaching movements^16–18^. Previous studies utilizing UCM have revealed coordinated patterns of upper limb joints that contribute to consistent and stable performance during motor tasks. To investigate kinetic synergies, which have received limited attention in previous studies, we incorporated a mathematical model based on the Jacobian matrix. The Jacobian matrix enables us to explore the relationship between joint torques and end-effector forces^27^, providing insights into the kinetic aspects of motor synergies. By combining both kinematic and kinetic assessments, our study provides a comprehensive understanding of motor synergies in individuals with stroke.

A forward-kinematics model was developed using modified Denavit–Hartenberg (DH) parameters defined in Table 1, where the three joints rotations are shoulder horizontal adduction (${\theta }_{s}$), elbow flexion (${\theta }_{e}$), and wrist flexion (${\theta }_{w}$), and the segment lengths are ${a}_{UA}$, ${a}_{FA}$ and ${a}_{H}$ for upper arm (from the shoulder joint center to the elbow joint center), forearm (from the elbow joint center to the wrist joint center) and the hand (from the wrist joint center to the middle finger tip), respectively shown as Fig. 2b. Based on the modified DH parameters, the transformation matrix is given by

**Table 1:** Modified Denavit–Hartenberg parameters.

Joint	${\alpha }_{i-1}$	${a}_{i-1}$	${d}_{i}$	${\theta }_{i}$
1	0	0	0	${\theta }_{s}$
2	0	${a}_{UA}$	0	${\theta }_{e}$
3	0	${a}_{FA}$	0	${\theta }_{w}$
4	0	${a}_{H}$	0	0

1 $${T}_{i-1,i}=\left[\begin{array}{cccc}\mathrm{cos}{\theta }_{i}& -\mathrm{sin}{\theta }_{i}& 0& {a}_{i-1}\\ \mathrm{sin}{\theta }_{i}\mathrm{cos}{\alpha }_{i-1}& \mathrm{cos}{\theta }_{i}\mathrm{cos}{\alpha }_{i-1}& -\mathrm{sin}{\alpha }_{i-1}& -{d}_{i}\mathrm{sin}{\alpha }_{i-1}\\ \mathrm{sin}{\theta }_{i}\mathrm{sin}{\alpha }_{i-1}& \mathrm{cos}{\theta }_{i}\mathrm{sin}{\alpha }_{i-1}& \mathrm{cos}{\alpha }_{i-1}& {d}_{i}\mathrm{cos}{\alpha }_{i-1}\\ 0& 0& 0& 1\end{array}\right]$$

The forward kinematics is obtained by computing the overall matrix of transformation from the base frame to the tip of the end-effector.

A forward-kinematics model of the end-effector position as a function of joint angles was obtained from first two elements in the last column of ${T}_{\mathrm{0,4}}$:

2 $$\left[\begin{array}{c}{P}_{x}\\ {P}_{y}\end{array}\right]=\left[\begin{array}{c}{f}_{1}({\theta }_{s},{\theta }_{e}, \, {\theta }_{w}\text{)}\\ {f}_{2}({\theta }_{s},{\theta }_{e}, \, {\theta }_{w}\text{)}\end{array}\right]$$

where ${f}_{1}={ a}_{UA}\mathrm{cos}\left({\theta }_{s}\right)+{a}_{FA}\mathrm{cos}\left({\theta }_{e}\right)+{a}_{H}\mathrm{cos}\left({\theta }_{w}\right)$ and ${f}_{2}={a}_{UA}\mathrm{sin}({\theta }_{s})+{a}_{FA}\mathrm{sin}({\theta }_{e})+{a}_{H}\mathrm{sin}({\theta }_{w})$, and ${P}_{x}$ and ${P}_{y}$ represent the coordinates of the end-effector position.

The transformation matrix T in Eq. (1) represents the homogeneous transformation between two consecutive frames based on the modified DH parameters. It captures both the rotation and translation between the (i − 1)-th and i-th coordinate frames. The forward kinematics model, as given in Eq. (2), utilizes the elements of the transformation matrix T to obtain the end-effector position as a function of the joint angles.

Using the Jacobian matrix (J), a linearized task equation, position of the end-effector as a function of joint angles was computed. The null space of J represents the changes in elemental variables that do not lead to a change in the performance variable, referred to as task-irrelevant space, whereas the orthogonal component of J represents the changes in elemental variables that do lead to a change in the performance variable, referred to as task-relevant space. The basis vectors, $\upvarepsilon$, for the null space was calculated such that $\mathrm{J\varepsilon }=0$.

Two types of variability, the deviations from the average trajectories in joint space were calculated as a variance of joint angles projected onto task-irrelevant space, ${\theta }_{TIR}$, and task-relevant space, ${\theta }_{TR}$, respectively.

3 $${TIV}_{\theta }=\frac{1}{n\cdot {dim}_{TIR}}\sum_{i=1}^{n}{\left|{\theta }_{TIR}\right|}^{2}$$

and

4 $${TRV}_{\theta }=\frac{1}{n\cdot {dim}_{TR}}\sum_{i=1}^{n}{\left|{\theta }_{TR}\right|}^{2}$$

where ${\theta }_{TIR}=\upvarepsilon {\upvarepsilon }^{T}\left(\theta -\overline{\theta }\right),$ ${\theta }_{TR}=\left(\theta -\overline{\theta }\right)-{\theta }_{TIR}$, $n$ is the number of trials (n = 10) , ${dim}_{TIR}$ and ${dim}_{TR}$ are dimensions of task-irrelevant space (${dim}_{TIR}={dim}_{joint}-{dim}_{task}$), and task-relevant space (${dim}_{TR}={dim}_{joint}-{dim}_{TIR}$), respectively. Note that ${dim}_{joint}$ and ${dim}_{task}$ are the number of joints (= 3) and number of tasks (= 2), respectively.

We also quantified kinetic synergistic patterns of UE. Using the Jacobian matrix calculated above, a linearized task equation, force of the end-effector as a function of joint torques was computed.

5 $$\left[\begin{array}{c}{F}_{x}\\ {F}_{y}\end{array}\right]={J}^{+}\left[\begin{array}{c}{\tau }_{s}\\ {\tau }_{e}\\ {\tau }_{w}\end{array}\right]$$

where ${J}^{+}$ is a pseudoinverse,${J}^{+}={\left(J\cdot {J}^{T}\right)}^{-1}\cdot J$ and ${\tau }_{s}$, ${\tau }_{e}$, and ${\tau }_{w}$ represent the joint torques associated with the shoulder, elbow, and wrist, respectively.

The basis vectors, $\upvarepsilon$, for the null space of ${J}^{+}$ was calculated such that ${J}^{+}\upvarepsilon =0$. The variabilities, the deviations from the average trajectories in joint space are calculated as a variance of joint torques projected onto task-irrelevant space, ${\tau }_{TIR}$, and task-relevant space, ${\tau }_{TR}$, respectively.

6 $${TIV}_{\tau }=\frac{1}{n\cdot {dim}_{TIR}}\sum_{i=1}^{n}{\left|{\tau }_{TIR}\right|}^{2}$$

and

7 $${TRV}_{\tau }=\frac{1}{n\cdot {dim}_{TIR}}\sum_{i=1}^{n}{\left|{\tau }_{TR}\right|}^{2}$$

where ${\tau }_{TIR}=\upvarepsilon {\upvarepsilon }^{T}\left(\tau -\overline{\tau }\right)$ and ${\tau }_{TR}=\left(\tau -\overline{\tau }\right)-{\tau }_{TIR}$, ${dim}_{TIR}$ and ${dim}_{TR}$ are dimensions of task-irrelevant space (${dim}_{TIR}={dim}_{joint}-{dim}_{task}$), and task-relevant space (${dim}_{TR}={dim}_{joint}-{dim}_{TIR}$), respectively.

In the UCM analysis^16–18^, it was necessary to normalize the movement trajectories in time between trials. To achieve this, we normalized the data by expressing the time-course of movement data as a percentage of the total duration of each trial through resampling. This normalization approach allowed us to align and compare the movement trajectories across trials, even when they were not performed in the exact same duration.

The index of synergy (*IS*) was computed to quantify the degree of coordination and synergy among the joint variables in maintaining the target trajectory in task space. *IS* provides a measure of how effectively the joint variables work together to achieve the desired movement^18,25,28^. Specifically, ${IS}_{\theta }$ represents the coordination of joint angles, while ${IS}_{\tau }$ represents the coordination of joint torques. The calculation of IS is based on the ratio between the variances of joint variables. We define ${TRV}_{\theta }$ and ${TRV}_{\tau }$ as the variances of joint variables that affect changes in the end-effector (i.e., fingertip) trajectories of position and force, respectively. On the other hand, ${TIV}_{\theta }$ and ${TIV}_{\tau }$ represent the variances of joint variables that do not affect the end-effector trajectories. To calculate the index of synergy for joint angles (${IS}_{\theta }$), we use the following formula:

8 $${IS}_{\theta }=\frac{{TIV}_{\theta }-{TRV}_{\theta }}{{TIV}_{\theta }+{TRV}_{\theta }}$$

Similarly, the index of synergy for joint torques (${IS}_{\tau }$) is calculated using:

9 $${IS}_{\tau }=\frac{{TIV}_{\tau }-{TRV}_{\tau }}{{TIV}_{\tau }+{TRV}_{\tau }}$$

Higher *IS* values, where ${TIV}_{\theta }$ or ${TIV}_{\tau }$ are greater than ${TRV}_{\theta }$ or ${TRV}_{\tau }$ respectively, indicate stronger coordination and synergy among the joint variables. This suggests that the variances of joint angles or torques are coordinated in such a way as to minimize the variability of the end-effector trajectories across trials. Conversely, lower *IS* values suggest less effective coordination. By analyzing the index of synergy, we gain valuable insights into the coordination and synergy of joint variables during upper limb movements, providing a quantitative measure of motor synergy. This information helps us better understand the underlying mechanisms and control strategies involved in the execution of motor tasks.

### Statistical analysis

Statistical analysis was conducted using two-way repeated measures ANOVA with the main factors of Group (control group versus stroke group) and Movement (inward versus outward). The dependent variables, including ${TIV}_{\theta }$, ${TRV}_{\theta }$, ${TIV}_{\tau }$, and ${TIV}_{\tau }$, were log-transformed to correct for a non-normal distribution, as suggested by previous studies^10,29^. The level of statistical significance was set at p = 0.05.

## Results

Figure 2c depicts the end-effector trajectories as well as individual joint angles and torques over 10 repetitions during the reaching task from a representative subject in each group. This visualization allows for a comprehensive examination of the variations in shoulder, elbow, and wrist angles and torques throughout the 10 repetitions. The observed joint angle and torque patterns provide insights into the motor control strategies employed by the participants in both the control and post-stroke groups. Notably, the findings reveal distinct patterns of joint coordination and motor output between the two groups, indicating potential alterations in motor control mechanisms following stroke. The detailed analysis of individual joint angles and torques, in conjunction with the end-effector trajectories, contributes to our understanding of the motor impairments and adaptations observed in post-stroke individuals during reaching movements. All statistical results for the 2-way ANOVA of kinematic and kinetic synergies, as well as the final position error, were provided in Table 2.

**Table 2:** Statistical results for synergy and final position error.

Measure		Group	Movement/Target	Interaction
Kinematics	${IS}_{\theta }$	F_1,48_ = 18.365, *p* < **0.001**	F_1,48_ = 0.244, p = 0.624	F_1,48_ = 0.001, *p* = 0.971
	${TIV}_{\theta }$	F_1,48_ = 5.321, *p* = **0.002**	F_1,48_ = 0.485, p = 0.490,	F_1,48_ = 0.002, *p* = 0.964,
	${TRV}_{\theta }$	F_1,48_ = 0.074, *p* = 0.787	F_1,48_ = 0.008, p = 0.931	F_1,48_ < 0.001, *p* = 0.994
Kinetics	${IS}_{\tau }$	F_1,48_ = 11.566, *p* = **0.001**	F_1,48_ = 0.244, p = 0.623	F_1,48_ = 4.224, *p* = **0.045**
	${TIV}_{\tau }$	F_1,48_ = 26.181, *p* < **0.001**	F_1,48_ = 0.188, p = 0.667	F_1,48_ = 2.992, *p* = 0.090
	${TRV}_{\tau }$	F_1,48_ = 14.037, *p* < **0.001**	F_1,48_ = 0.016, p = 0.899	F_1,48_ = 0.001, *p* = 0.974
Final position error		F_1,48_ = 5.063, *p* = **0.029**	F_1,48_ = 8.132, p = 0.07	F_1,48_ = 4.664, *p* = **0.036**

Significant values are in [bold].

### Kinematic synergy

**Figure 3:**
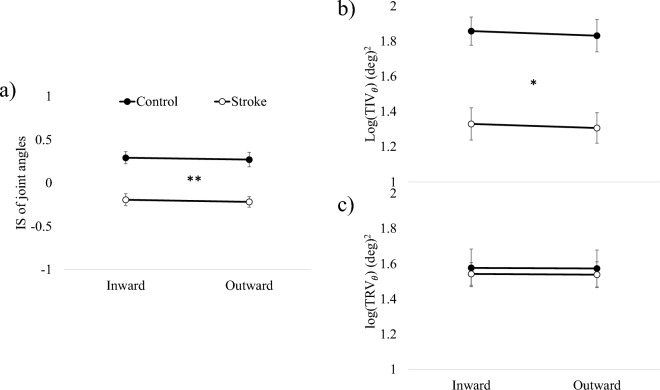
Index of synergy (*IS*) (**a**), logarithm of the task-relevant variability (*TIV*_*θ*_) (**b**) and task-irrelevant variability (*TRV*_*θ*_) (**c**) of joint angle were compared between the stroke group and the control group. *IS* of the stroke group was significantly lower than that of the control group. The decreased IS in Stroke group was main attributed to the decreased *TIV*_*θ*_ as compared to the control group. The asterisk indicates a significant difference (* < 0.05, ** < 0.001) between groups. Error bars represent standard error of the mean (SEM) across subjects.

${IS}_{\theta }$, ${TIV}_{\theta }$ and ${TRV}_{\theta }$ were depicted in Fig. 3. The stroke group exhibited significantly lower ${IS}_{\theta }$ compared to the control group. This finding was supported by a 2-way repeated measures ANOVA, showing a significant main effect of Group (F_1,48_ = 18.365; *p* < 0.001), no significant main effect of Movement (F_1,48_ = 0.244; *p* = 0.624), and no significant interaction Group $\times$ Movement (F_1,48_ = 0.001; *p* = 0.971). Furthermore, the stroke group had significantly lower ${TIV}_{\theta }$ compared to the control group, while ${TRV}_{\theta }$ did not differ between the two groups. These results were supported by a 2-way repeated measures ANOVA, revealing a main effect of Group (${TIV}_{\theta }$: F_1,48_ = 5.321; *p* = 0.002, ${TRV}_{\theta }$: F_1,48_ = 0.074; *p* = 0.787), a main effect of Movement (${TIV}_{\theta }$: F_1,48_ = 0.485; *p* = 0.490, ${TRV}_{\theta }$: F_1,48_ = 0.008; *p* = 0.931), and no significant interaction Group × Movement (${TIV}_{\theta }$: F_1,48_ = 0.002; *p* = 0.964, ${TRV}_{\theta }$: F_1,48_ < 0.001; *p* = 0.994). These results suggest that post-stroke patients exhibited lower variabilities in task-irrelevant joint angles without significantly affecting changes in end-effector trajectories compared to control subjects. Moreover, the variability of task-relevant joint angles, which is mathematically equivalent to the variability of end-effector trajectories across trials and affects end-effector trajectories, was found to be similar between the stroke and control groups.

### Kinetic synergy

**Figure 4:**
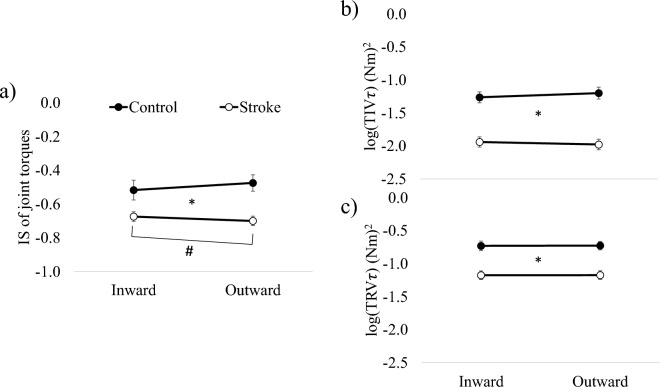
Index of synergy (*IS*) (**a**), logarithm of the task-relevant variability (*TIV*_*τ*_) (**b**) and task-irrelevant variability (*TRV*_*τ*_) (**c**) of joint angle were compared between groups during inward and outward movements. *IS, TIV*_*τ*_ and *TRV*_*τ*_ of the stroke group was significantly lower as compared to the control group. In addition, *IS* of the stroke group in outward movement was significantly lower as compared to inward movement while *IS* of the control group remained unchanged between movements. The asterisk (*) and the sharp (#) indicate a significant difference (* < 0.05, # < 0.05) between groups, and movement directions, respectively. Error bars represent SEM across subjects.

Figure 4 presents ${IS}_{\tau }$, ${TIV}_{\tau }$ and ${TRV}_{\tau }$. A 2-way repeated measures ANOVA was conducted to examine the effects of Group and Movement on ${IS}_{\tau }$. The analysis revealed a significant main effect of Group (F_1,48_ = 11.566; *p* = 0.001), indicating that ${IS}_{\tau }$ of joint torques in the stroke group was significantly lower than that of the control group. However, there was no significant main effect of Movement (F_1,48_ = 0.244; *p* = 0.623). Importantly, a significant interaction effect of Group and Movement was observed (F_1,48_ = 4.224; *p* = 0.045). Regarding ${TIV}_{\tau }$ and ${TRV}_{\tau }$, both measures showed significant main effects of Group (${TIV}_{\tau }$: F_1,48_ = 26.181, *p* < 0.001; ${TRV}_{\tau }$: F_1,48_ = 14.037, *p* < 0.001), indicating that the stroke group exhibited significantly lower ${TIV}_{\tau }$ and ${TRV}_{\tau }$ compared to the control group. However, there was no significant main effect of Movement for either ${TIV}_{\tau }$ (F_1,48_ = 0.188, *p* = 0.667) or ${TRV}_{\tau }$ (F_1,48_ = 0.016, *p* = 0.899). Additionally, the interaction effect of Group and Movement was not significant for both ${TIV}_{\tau }$ (F_1,48_ = 2.992, *p* = 0.090) and ${TRV}_{\tau }$ (F_1,48_ = 0.001, *p* = 0.974). Overall, the results indicate that stroke patients exhibited a lower degree of synergy in joint torque production compared to the control subjects, as evidenced by lower ${IS}_{\tau }$ values. Moreover, the deterioration in synergy was more pronounced during the outward movement in the stroke group. Additionally, both ${TIV}_{\tau }$ and ${TRV}_{\tau }$ were significantly lower in the stroke group, reflecting reduced variability in joint torques compared to the control group.

### Final position error

**Figure 5:**
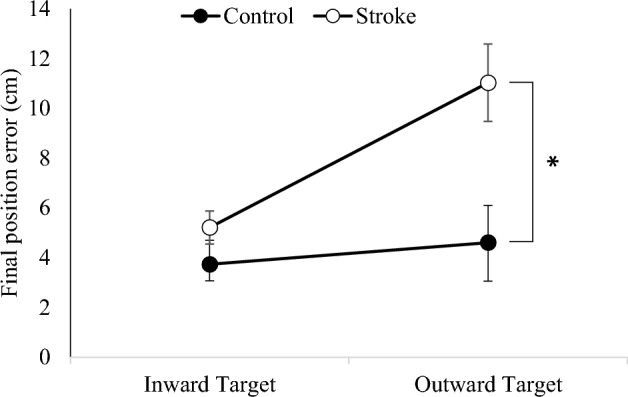
Comparison of final position errors for the Inward Target and Outward Target between the stroke group and the control group. There was no significant difference between groups for the Inward Target. However, a significant interaction was observed between Group and Target. The final position error in the stroke group was significantly higher for the Outward Target compared to the control group (* *p* < 0.05). Error bars represent the standard error of the mean (SEM) across subjects.”

The final position error, representing the distance between the hand position and the target, was calculated and illustrated in Fig. 5. It was observed that the stroke group encountered more difficulty in reaching the Inward Target compared to the Outward Target. These results were substantiated by a 2-way repeated measures ANOVA, which revealed a significant main effect of Group (F_1,48_ = 5.063; *p* = 0.029), a significant main effect of Target (F_1,48_ = 8.132; *p* = 0.07), and a significant Group $\times$ Target interaction (F_1,48_ = 4.664; *p* = 0.036). Specifically, the final position error for the Inward Target was significantly higher in the stroke group compared to the control group, while no significant difference was found in the final position error for the Outward Target compared to the control group. These findings highlight the specific challenges faced by the stroke group in reaching the Inward Target.

### Correlation with clinical score

**Figure 6:**
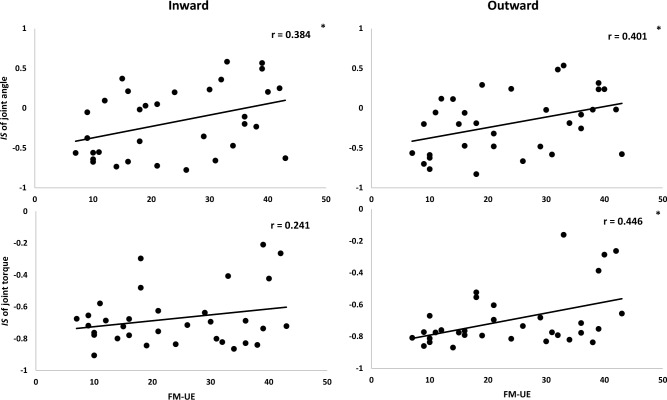
Scatter plot and correlation for IS and FMA-UE. During inward and outward movements, IS of joint angles were significantly correlated with FMA-UE (upper panel). R-scores during inward and outward were 0.384 and 0.401, respectively. IS of joint torques were significantly correlated with FMA-UE during outward movement but it was not significantly correlated during inward movement (lower panel). R-scores during inward and outward were 0.241 and 0.446, respectively.

We analyzed relationship of coordination patterns quantified as *IS*_*τ*_ to the UE function clinically evaluated with FMA-UE. We tested these correlations for kinematic and kinetic aspects during inward and outward movements, for four total correlations (Fig. 6). Kinematics *IS*_*θ*_ during both inward and outward movements were positively correlated with the FMA-UE score, with higher correlation coefficient (inward: r = 0.384, outward: r = 0.401) during outward movements. Kinetic *IS*_*τ*_ was positively correlated with the FMA-UE during outward movements. However, during inward movements, it was not significantly correlated with the FMA-UE.

## Discussion

In this study, we investigated whether stroke survivors retain the ability to coordinate multiple joints of the arm during a reaching task. We hypothesized that individuals post stroke would demonstrate deteriorated synergistic patterns both in kinematics and kinetics as compared to healthy control. We found decreased indices of kinematic and kinetic synergies in stroke survivors as compared to the control group, supporting the hypothesis. The reduction of synergy indices of joint angles was mainly attributed to the lower *TIV*_*θ*_ as compared to the control group, while the reduction of synergy of joint torque was due to the lower *TIV*_*τ*_ and higher *TRV*_*τ*_. These results suggest that stroke induces abnormal coordination patterns of UE movements with altered structure of variabilities in the multiple-joint movements and torques.

We also hypothesized that the expected impairment of synergy in stroke survivors would be more severe during outward movement as compared to inward movement. We found that kinetic synergy in the stroke group during outward movement was lower as compared to inward movement while kinetic synergy in the control group remained unchanged between movement directions. Our findings were consistent with a previous study^30^ that found stroke survivors tend to lean their body forward to extend the reach of the arm instead of using elbow extension. This compensative trunk movement during arm reaching task, along with our findings is possibly tied to the increased coupling in spastic upper extremity^31,32^. Thus, increased hyperexcitability of the stretch reflex after stroke may play a role in the formation of abnormal coordination patterns especially during UE outward movements.

We found that the stroke group had lower index of both kinematic and kinetic synergies as compared to the control group. This result demonstrates that individuals with stroke have a lesser ability to utilize synergic joint combinations to accomplish the reaching task. Our finding of decreased kinematic synergy is consistent with the clinical observation on individuals with stroke who often demonstrates fewer and less flexible patterns of joint coupling leading to difficulty in isolating joint motions^33^. In addition, the finding of the decreased kinetic synergy indicates that the stroke group had abnormal kinetic coordination across the multiple joints across the arm, possibly tied to the increased coupling in spastic upper extremity^31,32^. Taken together, our results confirm the common clinical observation that stroke survivors have limited, and different patterns of joint couplings compared with age-matched control persons. In accordance with previous conclusions that individuals with stroke demonstrate `disrupted inter-joint coordination' during reaching^34^, our results in the current study suggested that stroke leads to deficits on the CNS’s ability in utilizing the multiple DoFs of the UE for successful completion of a reaching task.

We found increased *TRV* of joint torques in the stroke group compared to the control group, indicating greater variability in hand force throughout the reaching movement for individuals with stroke. This finding suggests that the increased coupling in the spastic upper extremity may disrupt the inter-joint coordination of the UE, leading to higher variability in force production. However, there was no significant difference in *TRV* of joint angles between the two groups, indicating similar variability in hand trajectories. This result contradicts the initial statement regarding increased *TRV* of joint angles. It is important to note that previous studies on changes in kinematic motor synergies in individuals with stroke have yielded inconsistent findings^16–18^. This inconsistency suggests that kinematic quantification may be less sensitive in detecting motor deficits related to motor synergies compared to kinetic quantification. Additionally, we observed a decreased *TIV* of joint angles in the stroke group, indicating that individuals with stroke had less variability in individual joint angles, resulting in a more consistent hand position throughout the reaching movements. Previous studies have suggested that higher *TIV* values are beneficial in dealing with unexpected perturbations^35^, fatigue^36^, and secondary tasks^37^, indicating greater flexibility in utilizing degrees of freedom to accomplish a task. Our results align with the idea that stroke diminishes such flexibility in UE movement, supporting the notion of impaired inter-joint coordination.

In addition to this, we found that abnormal coordination patterns in the stroke group positively correlated with the FMA-UE, indicating that the worse coordination patterns are, the weaker the UE function measured by FMA-UE. The positive correlation was stronger during outward movement as compared to during inward movement. These results suggest that the ability to accomplish the UE reaching task with abundant movement solutions (i.e., greater *TIV*) may be closely related to motor deficits of UE motor functions. The findings in the current study were consistent with the previous findings that shows stroke survivors with weaker motor synergy tend to have severe motor deficits measured in FMA scores^3,38^.

Analysis of joint coordination patterns using the UCM may provide a foundation for the development of better identification of motor impairment and individualized therapeutic strategies of impaired motor coordination of UE. Previous studies found that abnormal kinematic joint coupling^13^ and kinetic coupling^39^ can be improved by targeted motor training. Therefore, it may be possible to develop training protocols that more directly focus on abnormal kinematic and kinetic synergistic patterns. In addition, assistive approaches, such as functional electrical stimulation and cortically driven prosthetic devices, are potentially useful for restoring motor synergies. Finally, it may be possible to design longitudinal studies to track the development of abnormal motor synergies during recovery. When combined with functional brain imaging, these studies may provide new insight to neural reorganization following stroke and help shape the nature and timing of acute and subacute therapeutic interventions.

In summary, the results of the present study demonstrated that individual with stroke had impaired ability in utilizing the multiple DoFs of the UE for successful completion of a reaching task as compared to age-matched control group. This impaired ability leads to kinematic and kinetic abnormal coordination patterns of the UE during a target-reaching task. Kinematically, individual with stroke showed higher trial-to-trial variability of hand’s trajectories and decreased flexibility of joint configurations as compared to age-matched control group. Kinetically, individual with stroke had higher trial-to-trial variability of hand’s force. Our results suggested that stroke may lead to motor deficits in formation of synergistic patterns of UE. Our findings in abnormal synergistic patterns of UE would provide better identification of motor impairment and planning for impairment-specific treatment.

## Data Availability

The datasets generated during and/or analyzed during the current study are available from the corresponding author on reasonable request.
